# Interleukin-6, CD8^+^ T cells, and Alzheimer’s disease: unraveling neuroimmune crosstalk via genetic and mechanistic insights

**DOI:** 10.3389/fnagi.2026.1767927

**Published:** 2026-03-23

**Authors:** Rui Xu, Weizheng Song, Xiaodong Zhang

**Affiliations:** Sixth People's Hospital of Chengdu, Chengdu, China

**Keywords:** Alzheimer’s disease, biomarkers, CD8^+^ T cells, immune-neuroendocrine interaction, interleukin-6, Mendelian randomization, neuroinflammation

## Abstract

Alzheimer’s disease (AD) is a neurodegenerative disorder with complex interplay between neuroinflammation and immune dysfunction. Interleukin-6 (IL-6), a pleiotropic cytokine, has emerged as a controversial player in AD pathogenesis, with conflicting roles reported in inflammation and neuroprotection. This review synthesizes genetic and mechanistic evidence linking IL-6 to AD, focusing on the mediating role of peripheral immune cells—particularly CD8^+^ T cell subsets like CD28^+^ CD45RA^−^ CD8br absolute counts (AC). We discuss how Mendelian randomization (MR) studies, including our recent work, have clarified causal relationships between IL-6, immune cell phenotypes, and AD risk. Additionally, we explore underlying mechanisms, such as IL-6-driven T cell activation, blood–brain barrier (BBB) modulation, and neuroinflammation resolution. Current controversies, including ethnic heterogeneity in genetic effects and the dual nature of IL-6 in systemic vs. central immunity, are highlighted. Finally, we address translational implications, such as immune cell-based biomarkers and targeted anti-inflammatory therapies, offering perspectives for future research.

## 1 Introduction: Alzheimer’s disease as a complex neuroimmune disorder

Alzheimer’s disease (AD), the most prevalent form of dementia affecting over 55 million people globally, is no longer viewed as a purely neurodegenerative disorder but as a complex neuroimmune disease influenced by age (Amelimojarad et al., 2024), sex (with females at higher risk) (Wingo et al., 2023; Ferretti et al., 2018), and genetic factors like the APOE ε4 variant (Haage and De Jager, 2022; Iannucci et al., 2021). Its core pathology involves amyloid-*β* (Aβ) plaques, tau neurofibrillary tangles, and neuroinflammation driven by activated microglia and astrocytes releasing pro-inflammatory cytokines, with emerging evidence highlighting the pivotal role of both innate and adaptive immune systems in disease progression (Liu et al., 2022; Onyango et al., 2021; Jorfi et al., 2023a). Innate immunity mediates chronic neuroinflammation via M1-polarized microglia (Zhong et al., 2024; Yao and Zu, 2020). Adaptive immune cells (T and B cells) play dual roles: Th1/Th17 cells aggravate inflammatory injury, whereas regulatory T cells and specific B-cell subsets exert neuroprotective effects (Jorfi et al., 2023b; Zhang et al., 2025). Furthermore, peripheral immune cells including monocytes, T cells, and B cells bridge systemic and central nervous system (CNS) inflammation by secreting cytokines that cross the blood–brain barrier (BBB), thereby activating neurotoxic glia and exacerbating Aβ and tau pathology (Zhang et al., 2025; Walker et al., 2023; Xiang et al., 2025).

Genetic studies link immune-related gene variants to AD susceptibility, while epidemiological data associate chronic infections and autoimmune conditions with increased disease risk. Monocytes infiltrate the brain to modulate Aβ clearance (Zhou et al., 2023), and T cell subsets exhibit protective or pathogenic effects in neuroinflammation (Xie et al., 2024), illustrating the bidirectional crosstalk between peripheral and CNS immunity that is further intertwined through pathways like the gut-brain axis (Gao et al., 2022), forming a multi-system disease network.

This review systematically synthesizes the current understanding of AD: it elaborates on the potential mechanisms by which Interleukin-6 (IL-6) and T cells drive AD occurrence and progression, clarifies their dual role in regulating AD-related neuroinflammation and neural function, and reveals their interactive relationships with key AD pathologies (e.g., Aβ plaques and tau tangles) within the neuroimmune network. This evolving knowledge has shifted therapeutic strategies from targeting isolated neural dysfunction to integrating immune modulation and neurorepair, with precision interventions emerging as promising non-invasive approaches.

## 2 Mendelian randomization: a robust tool for dissecting causal relationships in neuroimmunology

### 2.1 Core assumptions and analytical frameworks of Mendelian randomization

Mendelian randomization (MR) is a statistical approach that leverages genetic variants as instrumental variables (IVs) to infer causal relationships between exposures (e.g., cytokine levels, immune cell phenotypes) and outcomes (e.g., Alzheimer’s disease risk), with its core logic hinging on the random assortment of genetic variants during meiosis—mimicking the design of a randomized controlled trial to mitigate confounding biases inherent in observational studies.

MR relies on three critical assumptions for valid causal inferences: (1) Relevance: IVs (typically single nucleotide polymorphisms, SNPs) must be strongly associated with the exposure (e.g., SNPs tagging IL-6 production such as rs1800795 (Rai et al., 2021) or signaling such as IL-6R variants robustly correlating with circulating IL-6 levels or CD8^+^ T cell subset frequencies); (2) Independence, IVs must be independent of confounding factors influencing the outcome (e.g., age, obesity, comorbidities), as genetic variants fixed at conception are less likely to be confounded by environmental or lifestyle factors that obscure associations in observational studies; (3) Exclusion restriction: IVs must affect the outcome exclusively through the exposure (i.e., no horizontal pleiotropy) to ensure genetic variants influence AD risk only via their effect on IL-6 or CD8^+^ T cells rather than alternative pathways (Emdin et al., 2017). To address potential violations of these assumptions, sensitivity analyses (e.g., MR-Egger regression, Cochran’s Q test, leave-one-out analyses) are employed to detect and account for horizontal pleiotropy or heterogeneity, enhancing result robustness.

### 2.2 Application of MR in unraveling IL-6–CD8^+^ T cell–AD crosstalk

In exploring neuroimmune crosstalk in AD, MR enables multi-layered causal inference: Bidirectional MR, distinguishes causal directionality (e.g., verifying whether elevated IL-6 levels causally reduce AD risk rather than AD pathology increasing IL-6 levels, a reverse causality concern in observational studies); Multivariable MR (MVMR) quantifies the mediating role of intermediate variables (e.g., CD8^+^ T cell subsets)—as in our work, where MVMR revealed that CD28^+^CD45RA^−^CD8br absolute counts (AC) mediate 24.7% of IL-6’s protective effect against AD (Xu and Gao, 2025), clarifying the mechanistic link between IL-6 signaling and immune cell-mediated neuroprotection.

MR addresses critical limitations of traditional approaches in studying IL-6, CD8^+^ T cells, and AD: Resolving conflicting evidence: Observational studies often link elevated IL-6 to increased AD risk, while MR—capturing lifelong genetic effects—revealed that genetically predicted higher IL-6 levels associate with reduced AD risk (OR = 0.941, 95% CI: 0.899–0.985, *p* = 0.009), highlighting the pitfalls of single-time-point measurements; (2) Establishing causal pathways: Unlike epidemiological data, MR identifies whether IL-6 directly influences AD risk or acts via peripheral immune mediators (e.g., CD8^+^ T cells)—pivotal for prioritizing therapeutic targets; (3) Overcoming confounding: MR’s independence assumption minimizes biases from comorbidities (e.g., obesity or cardiovascular disease) to strengthen confidence in IL-6’s intrinsic role in AD pathogenesis.

MR analyses demonstrated that elevated IL-6 levels were significantly associated with reduced AD risk and increased CD28^+^CD45RA^−^CD8br AC cell levels, while higher CD28^+^ CD45RA^−^CD8br AC cell levels correlated with lower AD risk; MVMR confirmed these cells partially mediated IL-6’s protective effect (Xu and Gao, 2025). Additionally, MR analyses identified 5 protective immune cell traits (e.g., CD4 Treg AC) and 7 risk-increasing ones (e.g., IgD^−^ CD38^+^ high-expression cells), with causal links: elevated IgD^+^CD24^−^ AC cells, CD4^+^ leukocytes, CD4^+^CD8dim AC cells raised AD susceptibility (OR = 1.03, 1.08, 1.06; all *p* < 0.05) (Shen et al., 2024), while higher effector memory double negative (DN) T cells and DN absolute counts (AC) cells reduced risk (OR = 0.95, 0.93; all *p* < 0.05) (Liao et al., 2024). By integrating genetic rigor with mechanistic inquiry, MR bridges the gap between association and causality, providing a robust framework to dissect the complex interplay of neuroimmune factors in AD.

## 3 The dual role of IL-6 in Alzheimer’s disease: inflammation vs. neuroprotection

### 3.1 Context-dependent functions of IL-6: from neuroprotection to neurotoxicity

IL-6, a pleiotropic cytokine, exhibits context-dependent dual roles in AD. At low, short-term levels, it exerts neuroprotective effects (Wang et al., 2019; Fang et al., 2013): enhancing brain-derived neurotrophic factor (BDNF) secretion (Leal et al., 2017; Wertz et al., 2020), synaptic plasticity (Willis et al., 2020), and Aβ phagocytosis by microglia (Dempsey et al., 2017), promoting microglial repair phenotypes (Willis et al., 2020), maintaining BBB integrity (Choi et al., 2023), and regulating brain glucose metabolism (Escrig et al., 2020). These effects are crucial for preserving brain homeostasis and mitigating early AD-related pathology.

In contrast, chronic overactivation of IL-6 triggers neurotoxicity via the nuclear factor-κB (NF-κB) pathway (Brasier, 2010), fueling harmful neuroinflammation. Persistent IL-6 elevation correlates with increased Aβ deposition, tau hyperphosphorylation, and neuronal damage, exacerbating disease progression. Moreover, IL-6 genetic polymorphisms [e.g., rs1800795 (Rai et al., 2021)] are associated with AD risk (Papassotiropoulos et al., 2001) further supporting its role in balancing neuroimmunity.

### 3.2 Discrepancies between observational and mendelian randomization studies

Notably, IL-6, a pro-inflammatory cytokine, exhibits conflicting associations with AD across study designs: Observational studies frequently link elevated plasma/cerebrospinal fluid IL-6 levels to increased AD risk, proposing mechanisms such as neuroinflammation, Aβ deposition, and tau pathology to drive neurodegeneration (Bradburn et al., 2017; Licastro et al., 2003). In contrast, MR studies yield divergent findings: While some MR research identifies no significant causal effects on AD risk (Belbasis et al., 2025), our MR analyses report that genetically predicted higher IL-6 levels are associated with a reduced AD risk, partially mediated by CD28^+^CD45RA^−^CD8br AC cells (Xu and Gao, 2025).

These discrepancies stem from multiple factors: (1) Temporal mismatches: Single-time-point measurements in observational studies capture acute inflammation, masking IL-6’s long-term neuroprotective roles modeled in MR; (2) Confounding: Aging, obesity, or comorbidities distort observational associations; (3) Reverse causality: AD pathology may influence IL-6 levels, complicating observational interpretations; (4) Pleiotropy: IL-6’s dual roles (e.g., neuroprotective effects in acute injury) and genetic variants’ limited ability to capture tissue-specific effects (e.g., brain vs. systemic inflammation) contribute to inconsistencies (Belbasis et al., 2025). Collectively, these findings underscore the complexity of IL-6’s role in AD, highlighting the need to distinguish between systemic and CNS-specific IL-6 pathways.

## 4 IL-6 signaling pathway and CD8^+^ T cell subsets: mechanisms of neuroimmune crosstalk

### 4.1 IL-6-mediated JAK–STAT signaling in CD8^+^ T cell activation and differentiation

The IL-6 signaling pathway plays a central role in T cell activation and the differentiation of CD8^+^ T cells, primarily through the Janus kinase (JAK) - signal transducer and activator of transcription (STAT) pathway. When IL-6 binds to its receptor complex, it initiates a cascade that activates JAK1 and JAK2 kinases, which phosphorylate gp130. Phosphorylated gp130 recruits and phosphorylates STAT3, which dimerizes and translocates to the nucleus to regulate gene expression (Zhang J. et al., 2023; Xue et al., 2023).

IL-6 exerts distinct functions at different stages of CD8^+^ T cell development: (1) Priming phase: Enhances costimulatory signals and reprograms cellular metabolism to support proliferation (Brown et al., 2019); (2) Effector cell differentiation: Promotes cytotoxic function (Bottcher et al., 2014), inhibits apoptosis, and induces Th1/Th17-like polarization depending on the cytokine microenvironment (Yang et al., 2016); (3) Memory cell formation: Low or transient IL-6 exposure facilitates central memory T cell development, while high or persistent stimulation leads to effector memory or exhausted T cell generation (Feng et al., 2023; Daudelin et al., 2013).

### 4.2 Functional heterogeneity of CD8^+^ T cell subsets in neuroinflammation

CD8^+^ T cell subsets exhibit opposing functions in neuroinflammation: Cytotoxic CD8^+^ T cells eliminate infected or damaged cells by releasing perforin, granzymes, and pro-inflammatory cytokines (e.g., IFN-*γ*) (Shi et al., 2023; Zhao et al., 2018). However, excessive activation exacerbates tissue damage and neurodegeneration, as observed in AD and multiple sclerosis. In contrast, regulatory CD8^+^ T cells maintain immunological tolerance by secreting anti-inflammatory cytokines (e.g., IL-10, TGF-*β*) or via direct cell-surface interactions, protecting the CNS from collateral damage and promoting tissue repair (Cai et al., 2022).

### 4.3 IL-6–CD8^+^ T cell crosstalk in microglial polarization and Aβ/tau pathology

In animal models of AD, CD8^+^ T cells infiltrate the brain parenchyma around Aβ plaques, exerting complex effects on Aβ clearance and microglial activity (Feng et al., 2023). Some studies indicate that CD8^+^ T cells enhance Aβ clearance by promoting microglial phagocytosis (via direct cell–cell interactions or IFN-*γ* secretion to polarize microglia toward a pro-phagocytic state) (He et al., 2020). However, excessive infiltration leads to microglial overactivation, triggering chronic inflammation, harmful cytokine release, and reactive oxygen species (ROS) production (Wang et al., 2024)—impeding Aβ clearance and damaging neurons (Jorfi et al., 2023b).

**Figure 1:**
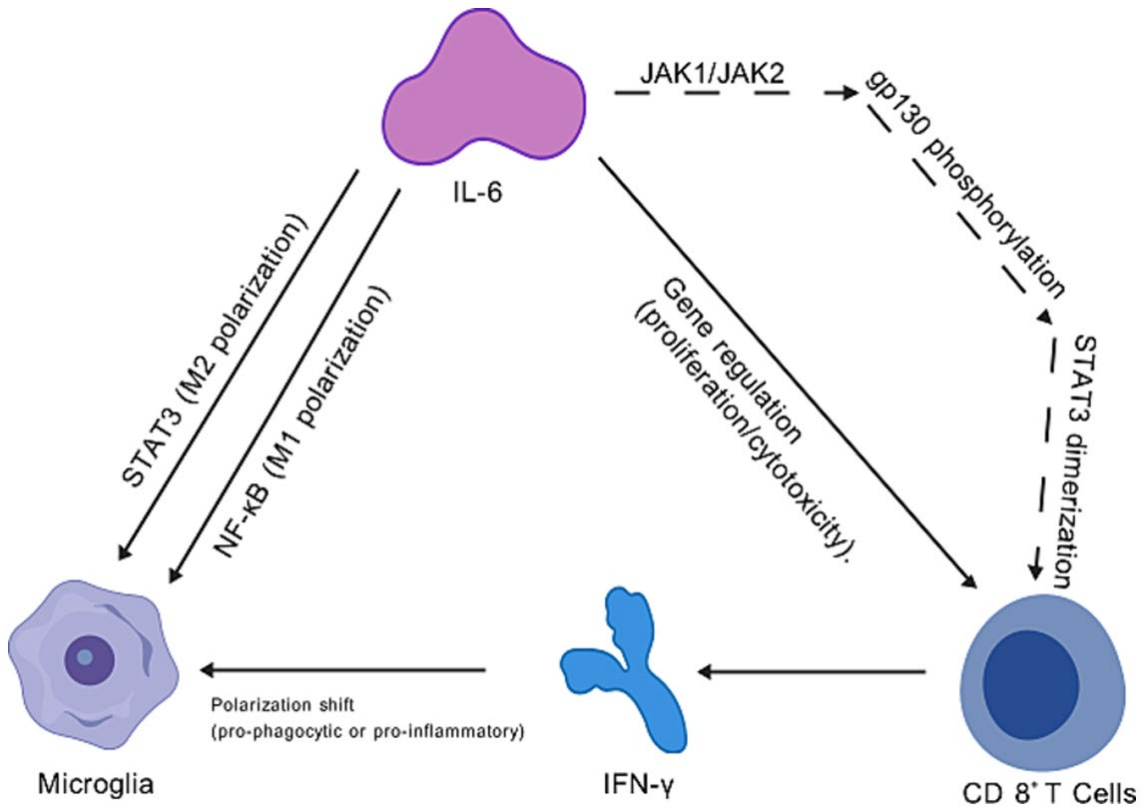
Cellular interactions regulated by IL-6 signaling.

Two main mechanisms underlie IL-6’s modulation of the neuroinflammatory environment: (1) IL-6 activates the JAK–STAT pathway in CD8^+^ T cells, enhancing their proliferation, cytotoxic function, and IFN-γ secretion (Jiang et al., 2021), thereby promoting neurotoxic aggregate clearance in collaboration with microglia (Garber et al., 2019; West et al., 2019); (2) IL-6 induces microglia to adopt an anti-inflammatory M2 phenotype via STAT3 signaling, reducing pro-inflammatory cytokine secretion (Jin et al., 2022). These interactions are fine-tuned by IFN-*γ*, which enhances IL-6-induced JAK1/JAK2-STAT3 activation in CD8^+^ T cells and modulates microglial responsiveness to IL-6 (Sun et al., 2023; Sun et al., 2017). The detailed diagrams can be referred to Figure 1, which were created using BioGDP.com (Jiang et al., 2025).

## 5 Stage-specific dynamics of IL-6–CD8^+^ T cell crosstalk across Alzheimer’s disease progression

### 5.1 Preclinical AD: protective crosstalk and homeostatic regulation

**Figure 2:**
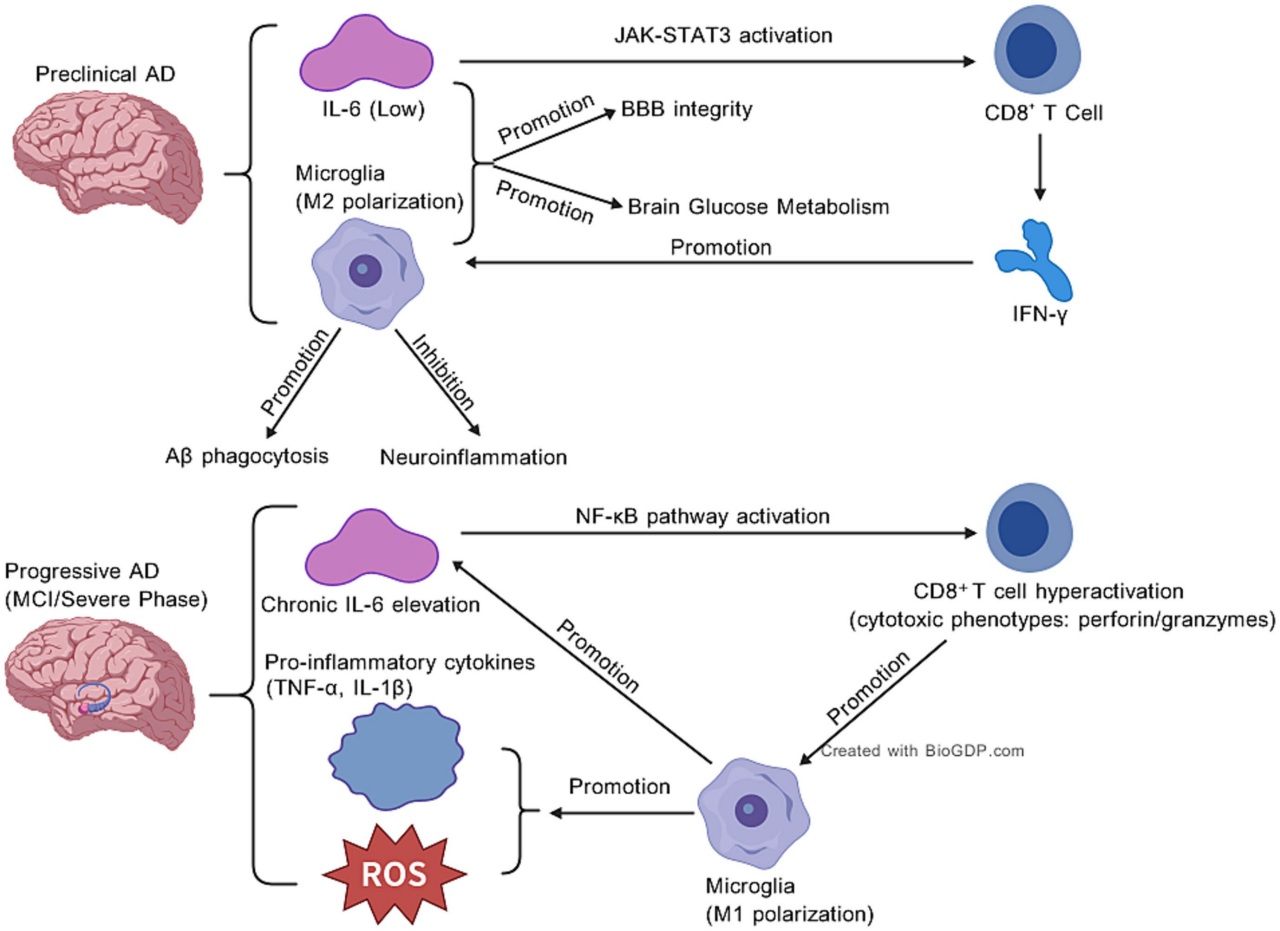
IL-6 and immune cell dynamics in preclinical vs. progressive AD.

In preclinical AD—characterized by early Aβ deposition without overt cognitive decline—IL-6 signaling favors protective CD8^+^ T cell responses. In this stage, IL-6 is constitutively secreted at low levels by astrocytes, resting microglia, and oligodendrocytes (Erta et al., 2012), with the former two responsible for basal production to maintain tissue homeostasis and the latter mediating modest secretion for synaptic maintenance; its expression is tightly controlled by the JAK–STAT3 feedback loop (Beurel and Jope, 2009) and selectively induced only by mild stress such as early Aβ deposition to drive protective microglial M2 polarization and CD8^+^ T cell-mediated Aβ clearance (Huang et al., 2025). MR studies (Xu and Gao, 2025) show genetically predicted higher IL-6 levels reduce AD risk, partially mediated by CD28^+^CD45RA^−^CD8br cells (linked to enhanced microglial Aβ phagocytosis). Here, IL-6 primes CD8^+^ T cells toward a moderately cytotoxic (Atsumi et al., 2009), IFN-*γ*-secreting phenotype that promotes Aβ clearance via microglial polarization (He et al., 2020). CD8^+^ T cell-derived IFN-γ reciprocally stimulates low-level IL-6 production from astrocytes, forming a homeostatic loop (Sun et al., 2023). Single-cell RNA sequencing supports this, revealing early STAT3 activation in peripheral CD8^+^ T cells—potentially driven by IL-6—that precedes clinical symptoms, suggesting a protective “priming phase” of crosstalk (Xu and Jia, 2021). The detailed diagrams can be referred to Figure 2, which were created using BioGDP.com (Jiang et al., 2025).

### 5.2 Progressive AD (MCI to moderate stage): transition to pathogenic feedforward loops

As AD progresses to mild cognitive impairment (MCI)—with emerging tau pathology and neuroinflammation—the protective balance shifts. IL-6 is now produced by a diverse array of cells, including infiltrating peripheral immune cells (CD8^+^ T cells, monocytes/macrophages) crossing the compromised BBB, overactivated M1 microglia and reactive astrocytes that serve as the dominant sources of chronic IL-6, and neurons undergoing stress-induced secretion in response to tau toxicity (Liu et al., 2024). Concomitantly, tight homeostatic control of IL-6 is lost: the JAK-STAT3 feedback loop is abrogated, the NF-κB pathway is robustly activated under the drive of pro-inflammatory cytokines such as TNF-α/IL-1β, leading to sustained IL-6 overproduction, and systemic IL-6 from comorbidities like obesity and cardiovascular disease further amplifies CNS IL-6 levels via BBB leakage (Sun et al., 2023; Sun et al., 2017).

Observational studies show elevated plasma IL-6 correlates with cognitive decline, reflecting a transition to chronic IL-6 overactivation (Economos et al., 2013). Persistent IL-6 signaling drives CD8^+^ T cell differentiation toward exhausted or hypercytotoxic phenotypes. In murine models, excessive CD8^+^ T cell infiltration around Aβ plaques triggers microglial overactivation and ROS release, impeding Aβ clearance (Jorfi et al., 2023b; Hu and Weiner, 2024). Conversely, hyperactivated CD8^+^ T cells amplify IL-6 production via direct microglial contact or IFN-*γ*-mediated astrocyte stimulation, creating a pro-inflammatory feedforward loop (Kedia et al., 2024). This stage-specific shift aligns with MR findings contrasting observational associations (elevated IL-6 linked to risk) and genetic causality (IL-6-associated protection), highlighting temporal mismatches in IL-6’s roles. The detailed diagrams can be referred to Figure 2, which were created using BioGDP.com (Jiang et al., 2025).

### 5.3 Severe AD: dysregulated crosstalk and exacerbated neurodegeneration

In moderate-to-severe AD—marked by extensive neurodegeneration and BBB disruption—bidirectional crosstalk becomes further dysregulated. Peripheral CD8^+^ T cells infiltrate the brain parenchyma more freely, and their interaction with IL-6 exacerbates tissue damage: cytotoxic CD8^+^ T cells release perforin and granzymes, damaging neurons; IL-6, now predominantly acting via the NF-κB pathway, sustains chronic neuroinflammation (Jorfi et al., 2023b; Shi et al., 2023). Regulatory CD8^+^ T cells—normally secreting IL-10 to limit damage—appear functionally impaired, possibly due to sustained IL-6 exposure inhibiting their differentiation (a mechanism observed in multiple sclerosis) (Zhao et al., 2018; Huseni et al., 2023).

### 5.4 Stage-specific clonal expansion and phenotypic shift of CD8^+^ T cells in AD

The stage-specific clonal expansion and phenotypic shift of CD8^+^ T cells are core adaptive immune features in AD, and the MR study further supplements the immunomodulatory role of CD8^+^ T cell subsets in AD. On one hand, human and preclinical studies have demonstrated that CD8^+^ T cells exhibit dual pathogenic and protective roles: in early AD and MCI, circulating CD8^+^ TEMRA cells increase with cognitive decline, infiltrate the CNS, accumulate around Aβ plaques, and undergo Epstein–Barr virus-associated clonal expansion with high expression of cytotoxic genes (Gate et al., 2020); while in late stages, they shift to an exhausted, tissue-resident phenotype to exert protective effects, with the CCL5-CCR5 axis identified as a potential early therapeutic target (Ohyagi et al., 2025). On the other hand, the MR study provides genetic evidence that the CD28^+^CD45RA^−^CD8br subset, a specific CD8^+^ T cell subpopulation, partially mediates the protective effect of IL-6 on reducing AD risk, highlighting that distinct CD8^+^ T cell subsets may exert divergent regulatory effects on AD pathogenesis. Collectively, these findings emphasize the heterogeneity and stage dependency of CD8^+^ T cell responses in AD: while certain subsets (e.g., CD8^+^ TEMRA) drive early neuroinflammation and amyloid pathology, others (e.g., CD28^+^CD45RA^−^CD8br) mediate protective signaling. This underscores the importance of dissecting CD8^+^ T cell subset-specific functions and their temporal dynamics for developing precision immunotherapies, and suggests that integrating genetic mediation evidence (e.g., IL-6-CD28^+^CD45RA^−^CD8br axis) with stage-specific phenotypic data may further refine AD immunomodulatory strategies (see Table 1).

**Table 1:** CD8+ T cell subsets in Alzheimer’s disease: phenotypic and functional.

Subset name	Surface markers	Cytokine/effector profile	Role in AD
Cytotoxic CD8^+^ T cells	CD45RA^+^, CD28^+^, CD8^+^, Perforin^+^, Granzyme^+^	Secretes IFN-γ, Perforin, Granzymes; Moderate cytotoxicity	- Protective: promotes Aβ clearance via microglial polarization (preclinical AD) - Pathogenic: excessive activation causes neuronal damage and exacerbates neuroinflammation (progressive/severe AD)
Regulatory CD8^+^ T cells	CD25^+^, Foxp3^+^, CD8^+^, PD-1^+^ (low expression)	Secretes IL-10, TGF-β; Inhibits pro-inflammatory cytokine release	Protective: maintains immunological tolerance, suppresses excessive neuroinflammation, and protects the central nervous system (CNS) from collateral damage
Effector memory CD8^+^ T cells (TEM)	CD45RA^−^, CD28^+^/^−^, CD8^+^, CCR7^−^	Secretes IFN-γ, TNF-α; Sustained effector function	- Protective: enhances A*β* clearance and microglial M2 polarization (preclinical AD) - Pathogenic: shifts to a hypercytotoxic phenotype in progressive AD, accelerating neurodegeneration
Terminal effector memory RA^+^ CD8^+^ T cells (TEMRA)	CD45RA^+^, CD28^−^, CD8^+^, CCR7^−^	Secretes high levels of Perforin, Granzymes; Moderate IFN-γ	Pathogenic: infiltrates the CNS, accumulates around Aβ plaques, and undergoes Epstein–Barr virus (EBV)-associated clonal expansion in early AD; Promotes amyloid pathology and cognitive decline
Exhausted CD8^+^ T cells	CD8^+^, PD-1^+^, Tim-3^+^, LAG-3^+^, CD45RA^−^/^+^	Reduced secretion of IFN-γ and TNF-α; Occasional increase in IL-10	Biphasic: - Early stage: Functional exhaustion limits excessive neuroinflammation - Late stage: impaired protective function fails to counteract pathological progression
Double negative (DN) effector memory T cells	CD4^−^, CD8^−^, CD45RA^−^, CCR7^−^	Secretes IL-10, TGF-β; Low cytotoxicity	Protective: reduces AD risk via anti-inflammatory effects; Mediates neuroimmune homeostasis
CD28^+^CD45RA^−^CD8br T cells	CD28^+^, CD45RA^−^, CD8br (high CD8 expression), CD8^+^	Secretes IFN-γ; Moderate cytotoxicity	Protective: mediates 24.7% of IL-6’s anti-AD effect; Enhances microglial Aβ phagocytosis and suppresses neuroinflammation

### 5.5 Modulation by comorbidities: obesity, cardiovascular disease, and autoimmune conditions

Comorbidities further modulate IL-6–CD8^+^ T cell crosstalk: (1) Obesity induces chronic systemic IL-6 elevation via adipose tissue, skewing CD8^+^ T cells toward a pro-inflammatory phenotype with reduced Aβ-clearing capacity—overriding IL-6’s neuroprotective genetic effects (Blaszczak et al., 2021; Ghazarian et al., 2015); (2) Cardiovascular disease, linked to BBB dysfunction, enhances CD8^+^ T cell infiltration into the brain, where they interact with IL-6-rich neuroinflammatory niches to accelerate tau pathology (Blair et al., 2015; Moreno-Valladares et al., 2020); (3) In autoimmune conditions (e.g., rheumatoid arthritis), chronic IL-6 blockade (e.g., tocilizumab) reduces peripheral CD8^+^ T cell cytotoxicity but may impair their protective roles in early AD, underscoring the need for stage-specific therapeutic timing (Chen et al., 2023). Collectively, these dynamics highlight IL-6–CD8^+^ T cell crosstalk as a “rheostat” tuned by disease stage and systemic inflammation.

## 6 Key challenges in translating neuroimmune insights to clinical practice

### 6.1 Translational gaps between peripheral and central nervous system immunity

A paramount challenge in AD research is bridging the gap between peripheral immune observations and CNS dynamics. Peripheral biomarkers (e.g., altered CD8^+^ T cell subsets or IL-6 levels) often fail to accurately reflect brain parenchyma immune processes. This discrepancy stems from the BBB (which tightly regulates immune cell infiltration) and divergent functional profiles of CNS-resident cells (e.g., microglia) versus infiltrating T cells. For instance, while peripheral CD8^+^ T cells in AD may exhibit heightened cytotoxicity, their brain entry and interaction with local immune microenvironments are governed by distinct chemokine gradients and BBB integrity—complicating the translation of peripheral findings to CNS pathology.

### 6.2 Ethnic heterogeneity in genetic associations and generalizability

Most AD genome-wide association studies (GWAS) predominantly focus on European populations, introducing ethnic bias that undermines the generalizability of genetic findings linking immune pathways to AD. A notable example is the IL-6 variant rs1800795 (Rai et al., 2021) (associated with elevated IL-6 levels), which shows marked ethnic variation in allele frequencies and effect sizes. Such disparities may obscure population-specific interactions between IL-6 signaling, CD8^+^ T cell function, and AD risk, underscoring the need for cross-ethnic validation to identify universally relevant biomarkers and therapeutic targets.

### 6.3 Unresolved questions on causal directionality of IL-6–CD8^+^ T cell crosstalk

A critical unresolved question is the causal directionality of IL-6–CD8^+^ T cell crosstalk. The reciprocal regulation between IL-6 and CD8^+^ T cells is not static but adapts to evolving AD neuropathology and systemic perturbations from comorbidities. While MR studies suggest IL-6’s protective role mediated by CD8^+^ T cells, the dynamic shifts in this crosstalk across disease stages (from protective to pathogenic) remain incompletely understood. Clarifying the temporal sequence of IL-6 and CD8^+^ T cell alterations is essential for defining optimal therapeutic windows.

## 7 Therapeutic implications and future research directions

### 7.1 Targeting IL-6 signaling: balancing neuroprotection and inflammation suppression

Modulating IL-6 signaling has emerged as a promising therapeutic avenue. Preclinical data suggest blocking IL-6 trans-signaling (via soluble IL-6 receptor) reduces neuroinflammation while preserving beneficial microglial A*β* phagocytosis (Pons-Espinal et al., 2024; Escrig et al., 2019). This approach avoids the pitfalls of global IL-6 blockade, which may abrogate neuroprotective effects in early AD. Additionally, selective JAK2 inhibitors could target IL-6-mediated pro-inflammatory pathways without disrupting protective STAT3-dependent signaling—offering a balanced strategy to mitigate neurotoxicity while preserving neuroprotection (Zhang W. et al., 2023).

### 7.2 Enhancing regulatory CD8^+^ T cell function for neuroprotection

Enhancing regulatory CD8^+^ T cell function represents another viable approach. For example, IL-10 or TGF-β mimics could suppress excessive cytotoxicity and promote tissue repair in the CNS (Levescot and Cerf-Bensussan, 2022). Strategies to expand CD28^+^CD45RA^−^CD8br cells (the mediator of IL-6’s protective effect) may also enhance Aβ clearance and microglial homeostasis—particularly in preclinical or early progressive AD. Combining these approaches with existing Aβ/tau-targeted therapies could synergistically address both neural degeneration and immune dysregulation.

### 7.3 Advanced research approaches to address mechanistic and translational gaps

Advancing this field requires targeted research efforts: (1) Dissect spatiotemporal dynamics: investigate IL-6 crosstalk with specific CD8^+^ T cell subsets (e.g., CD28^+^CD45RA^−^CD8br cells) across AD’s preclinical-to-progressive stages to clarify stage-specific regulatory mechanisms; (2) Leverage cutting-edge technologies: use single-cell multiomics, spatial transcriptomics (to resolve localization of IL-6-responsive T cells relative to Aβ plaques), and *in vivo* AD models to explore IL-6 signaling’s interplay with emerging pathologies (e.g., neuroinflammatory-amyloid/tau crosstalk); (3) Integrative multi-omics: combine genomics, immunomics, and neuroimaging to map spatiotemporal interactions between IL-6, CD8^+^ T cells, and CNS pathology (Naulaerts et al., 2023); (4) Cross-ethnic validation: Utilize cohorts like ADNI-GO to validate genetic associations, address GWAS biases, and develop personalized immunotherapies applicable to diverse populations.

### 7.4 Clinical application prospects: biomarkers and combination therapies

This research supports two key clinical applications: (1) Non-invasive biomarkers: Validate IL-6/STAT3 pathway-related molecules (e.g., phosphorylated STAT3 in peripheral immune cells) as prognostic AD biomarkers to complement existing A*β*/tau markers—enabling early disease detection and stage stratification; (2) Combination therapies: Evaluate the feasibility of combining IL-6 modulators (e.g., selective JAK2 inhibitors) with regulatory T cell enhancers to address current monotherapy limitations, leveraging synergistic effects on reducing neuroinflammation while preserving neuroprotection.

## 8 Conclusion

AD is increasingly recognized as a complex neuroimmune disorder, where IL-6 and immune cells (e.g., CD8^+^ T cells) exhibit stage-dependent dual pro-inflammatory and neuroprotective roles. MR studies have been pivotal in uncovering IL-6-mediated neuroprotection, partially mediated by CD28^+^CD45RA^−^CD8br T cells, and resolving conflicting evidence from observational studies. Key challenges include translational gaps between peripheral and central immunity, ethnic biases in genetic research, and unresolved questions on the causal directionality of IL-6–CD8^+^ T cell crosstalk.

Precision immunotherapies—targeting IL-6 trans-signaling, enhancing regulatory CD8^+^ T cell function, or modulating specific T cell subsets—emerge as viable therapeutic avenues, with stage-specific interventions critical to balancing neuroprotection and inflammation suppression. Future research leveraging advanced technologies (single-cell multiomics, spatial transcriptomics) and cross-ethnic cohorts will further refine our understanding of neuroimmune crosstalk, paving the way for holistic, mechanism-based therapies that address both neural degeneration and systemic inflammatory dysregulation in AD.

## Glossary

AC: Absolute Counts
AD: Alzheimer’s Disease
ADNI-GO: Alzheimer’s Disease Neuroimaging Initiative-GO
Aβ: Amyloid-*β*
APOE ε4: Apolipoprotein E ε4
APP/PS1: Amyloid Precursor Protein/Presenilin 1
BBB: Blood–Brain Barrier
BDNF: Brain-Derived Neurotrophic Factor
CAR T: Chimeric Antigen Receptor T Cells
cGAS-STING: Cyclic GMP-AMP Synthase/Stimulator of Interferon Genes
CNS: Central Nervous System
DN: Double Negative
ERK: Extracellular Regulated Protein Kinase
GWAS: Genome-Wide Association Studies
IFN-γ: Interferon-*γ*
IL-6: Interleukin-6
IL-1β: Interleukin-1β
IL-10: Interleukin-10
iPSC: Induced Pluripotent Stem Cells
JAK: Janus Kinase
JAK1: Janus Kinase 1
JAK2: Janus Kinase 2
MAPK: Mitogen-Activated Protein Kinase
MCI: Mild Cognitive Impairment
MR: Mendelian Randomization
MVMR: Multivariable Mendelian Randomization
NF-κB: Nuclear Factor-κB
NLRP3: NOD-Like Receptor Family Pyrin Domain Containing 3
PI3K: Phosphatidylinositol 3-Kinase
AKT: Protein Kinase B
PD-L1: Programmed Cell Death Ligand 1
ROS: Reactive Oxygen Species
SNP(s): Single Nucleotide Polymorphism(s)
STAT: Signal Transducer and Activator of Transcription
STAT3: Signal Transducer and Activator of Transcription 3
TEMRA: Terminal Effector Memory RA + T Cells
TGF-β: Transforming Growth Factor-β
TNF-α: Tumor Necrosis Factor-α
